# Computational Intelligence for Observation and Monitoring: A Case Study of Imbalanced Hyperspectral Image Data Classification

**DOI:** 10.1155/2022/8735201

**Published:** 2022-04-30

**Authors:** Debaleena Datta, Pradeep Kumar Mallick, Jana Shafi, Jaeyoung Choi, Muhammad Fazal Ijaz

**Affiliations:** ^1^School of Computer Engineering, Kalinga Institute of Industrial Technology, Deemed to Be University, Bhubaneswar 751024, India; ^2^Department of Computer Science, College of Arts and Science, Prince Sattam Bin Abdul Aziz University, Wadi ad-Dawasir 11991, Saudi Arabia; ^3^School of Computing, Gachon University, Seongnam-si 13120, Republic of Korea; ^4^Department of Intelligent Mechatronics Engineering, Sejong University, Seoul 05006, Republic of Korea

## Abstract

Imbalance in hyperspectral images creates a crisis in its analysis and classification operation. Resampling techniques are utilized to minimize the data imbalance. Although only a limited number of resampling methods were explored in the previous research, a small quantity of work has been done. In this study, we propose a novel illustrative study of the performance of the existing resampling techniques, viz. oversampling, undersampling, and hybrid sampling, for removing the imbalance from the minor samples of the hyperspectral dataset. The balanced dataset is classified in the next step, using the tree-based ensemble classifiers by including the spectral and spatial features. Finally, the comparative study is performed based on the statistical analysis of the outcome obtained from those classifiers that are discussed in the results section. In addition, we applied a new ensemble hybrid classifier named random rotation forest to our dataset. Three benchmark hyperspectral datasets: Indian Pines, Salinas Valley, and Pavia University, are applied for performing the experiments. We have taken precision, recall, *F* score, Cohen kappa, and overall accuracy as assessment metrics to evaluate our model. The obtained result shows that SMOTE, Tomek Links, and their combinations stand out to be the more optimized resampling strategies. Moreover, the ensemble classifiers such as rotation forest and random rotation ensemble provide more accuracy than others of their kind.

## 1. Introduction

In recent times, images have been one of the prime data sources. Hyperspectral images (HSIs) are currently in trend due to the enormous amount of information it captures in an earth surface scene. HS data are one type of data that can be used in various ways to develop human technology [1]. HSI refers to spectral imaging data acquired by satellites equipped with airborne spectrometers. The photographs take over specific earth surfaces, referred to as the scene, containing various land cover classes such as flora, concrete, and water bodies. Because each related land cover occupies a varied surface area, the number of pixels representing each class varies. However, HS data have various difficulties, including noise, quality and quantity of labeled data, dimensionality, and categorical sample imbalance [2]. Additionally, analyzing and interpreting HS data necessitate several processes, including denoising, lowering hyper-dimensionality, spectral unmixing, and, most critically, identifying land cover [3]. The classification of the imaging scene has been a preoccupation of professionals from the inception of hyperspectral data. Initially, they used statistics-based classifiers in conjunction with some preprocessing techniques. The categorization problem became easier to handle with breakthroughs in ML and the introduction of DL. It provides an excellent strategy to deal with the dataset's embedded concerns [4].

Imbalanced data refer to classification challenges in which the classes are not equally represented; the major class is the most common, while the minor class is the rarest [5]. Information security, medical imagery, bioinformatics, network intrusion, and fraud detection are just a few examples of real-world datasets that suffer from imbalanced classification [6]. The HSI dataset is also skewed since insufficient data instances belong to either of the class labels due to their different land area coverage. The sample distribution per class can range from a little imbalance to a severe imbalance, with few samples in the minor class and hundreds in the major class. Two basic criteria can also be used to demonstrate the HSI imbalance: (1) the minor class shortage of knowledge and (2) the imbalance ratio (ImbR), i.e., the ratio between the minor and major classes [7]. The mathematical formula is given as follows:

(1) $$\begin{array}{c} \text{ImbR}=\frac{\text{Number of class samples belonging to minor class}}{\text{Number of class samples belonging to major class}}. \end{array}$$

Due to its complicated data structure, HSI eventually confronts an imbalanced classification challenge. Unbalanced classifications complicate predictive modeling because most machine learning algorithms for classification were created with an equal number of samples per class in mind. As a result, models with lower prediction accuracy arise, particularly for the minor class, posing a problem because the minor class is frequently more significant than the major class. As a result, categorization errors are more probable to occur in the minor class than in the major class [8]. Because of the dataset's inherent complexity, learning from it demands new views, approaches, concepts, and methods for changing data. The most effective way to address the data imbalance is to resample the data instances to roughly equal proportions. The three types of sampling procedures employed are oversampling, undersampling, and hybrid sampling. Oversampling entails taking identical random data samples from the minor class, leading to overfitting. In contrast, undersampling entails removing random knowledge from the major class, resulting in information loss. There are also hybrid balancing strategies that use a collaborative effort between an oversampler and an undersampler to balance samples from each class in the same dataset [9]. The hybrid sampling method combines an oversampler and an undersampler that balances the dataset. After correcting the imbalance, a suitable model must be used to train the dataset. Logistic regression (LR), naive Bayes (NB), support vector machine (SVM), and tree-based classifiers are examples of benchmark machine learning algorithms suitable for moderately balanced datasets [10]. Ensemble learning methods have become increasingly prominent in the latest years. The primary objective of those systems is to increase performance by aggregating the findings of multiple weak classifiers. These systems employ a voting technique amongst all the weak classifiers to obtain the ultimate classification result [11]. A decision tree (DT) [12] is considered to be the most preliminary bagging technique. DT for each subset of the original dataset has been created individually. Finally, a voting mechanism was used to determine the final result among those DTs. Random forest (RF) [13] is the most widely utilized tree-based ensemble classifier based on the bagging approach for both classification and regression. The insensitivity of RF to spectral bands and its ability to handle missing and imbalanced data are two of its most enticing characteristics. It can also be used on noisy samples because it does not overfit the data easily [14]. The extra trees (ET) or extremely randomized trees [15] approach works by producing many unpruned decision trees from the training dataset. In contrast to classification, predictions are created for regression by taking averages of the prediction formed by the subordinate DTs, whereas for classification, the rule of majority voting is applied. Unlike RF and bagging, which build each DT using a bootstrap sample of the training dataset, ET fits each DT to the whole training dataset. Rotation forest (RoF) [16] algorithms outperform bagging on noise-free and imbalanced data. Compared with bagging and RF, RoF can achieve similar or more excellent results with fewer trees. Blaser and Fryzlewicz [17] proposed an ensemble of random and rotation forests in the name of random rotation ensemble forest (RREF). The random rotation efficiently creates a new coordinate system belonging to each base learner, increasing ensemble variety without sacrificing accuracy. Moreover, one significant distinction between the random rotation and the random projection is that rotations are reversible, meaning no information loss.

The premise that motivated us to pursue our research work is a broad analytical study of the prevailing resampling techniques and their impacts on the hyperspectral images, viz. oversampling, undersampling, and hybrid sampling. For oversampling, four useful techniques are selected, namely random oversampling (ROS) [18], synthetic minority oversampling technique (SMOTE) [19], borderline SMOTE (B-SMOTE) [20], and adaptive synthetic minority oversampling technique (ADASYN) [21]. Furthermore, four popular undersampling methods are studied, namely random undersampling (RUS) [22], Tomek Links (TLs) [23], neighborhood cleaning rule (NCL) [24], and edited nearest neighbor (ENN) [25]. Finally, two-hybrid sampling techniques are considered: SMOTETomek [26], a combination of SMOTE and TL, and SMOTEENN [27], a combination of SMOTE and ENN. These strategies are used to balance the dataset that will be learned through training. These balanced datasets are then passed to the classifiers as train inputs for categorizing the land covers in the HS scenes. In this work, we have used the eminent tree-based ensemble classifiers that are demonstrated for their compatibility with synthetically balanced, huge-dimensioned HSI datasets. The ensemble classifiers employed here are DT, ET, RF, RoF, and RREF. They are utilized to construct the entire comparison study where each of these classifiers is assessed using all the resampling techniques for individual datasets. The quality and performance of the model are evaluated over testing data using various metrics such as precision score, recall score, *F* score, overall accuracy, and Cohen kappa score.

Our work provides the following contributions:

- A comprehensive adaptation of conventional resampling algorithms to correct the hyperspectral datasets' substantial imbalance.
- A novel approach to classify hyperspectral images using efficient tree-based ensemble learning methods, i.e., the traditional tree-based techniques and the hybridized and modified forest methods to structure the classification model.
- An advanced comparative investigation of various oversampling, undersampling, and hybrid sampling strategies applied to hyperspectral datasets shows that they positively influence rectifying the major-minor sample imbalance.
- The innovative, thorough comparison of the applications of the included tree-based ensemble classifiers in categorizing the surface covers is captured in the HSI in terms of different performance assessment metrics. In addition, these classifiers are capable of learning joint spectral-spatial features of the balanced datasets.
- The comparative case study with all the resampling techniques used for HSI datasets can be carried forward to the other computational intelligence and monitoring applications with several types of datasets, especially in the area of medical imagery.
- The study provides more excellent knowledge for the researchers who deal with big data and voluminous imagery data that suffer due to imbalanced samples. The comparison depicted here benefits choosing the more appropriate strategy to work with different datasets and provides a better view of improvising those strategies.

The remaining paper is divided into the following categories: Section 2 describes previous work in the area of resampling for various imbalanced datasets, Section 3 depicts our research work's methodology, Section 4 illustrates the model evaluation and test results, and Section 5 provides the conclusion and also deliberates the research work's limitations and future scope.

## 2. Previous Works

**Figure 1:**
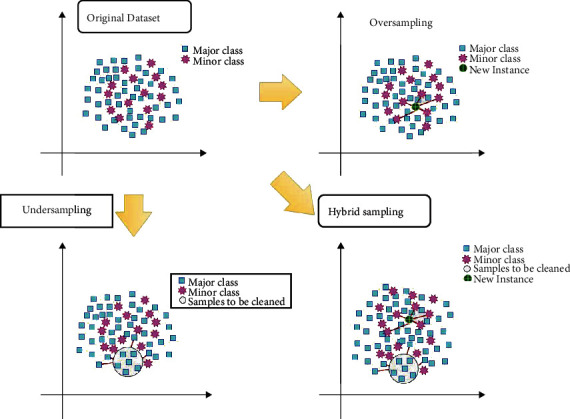
Representation of the resampling methods for balancing data.

Unbalanced data have produced numerous problems in the classification of hyperspectral images. Researchers have employed preprocessing techniques to deal with the issue of class imbalance in several application fields. Preprocessing approaches for the imbalanced class problem include data-driven methodologies, such as sampling. Oversampling, undersampling, and hybrid sampling are the three types of sampling. The suitable sampling technique to be chosen is determined by the dataset, the sample size of each class, and their ratio of imbalance (IR).  Figure 1 depicts the overall changes in the major and minor class samples in a dataset using three different sampling procedures. One class, i.e., the major class, is dominant over another class considered minor in the original dataset. In oversampling, the minor class samples are overpopulated to match the number of major class samples by creating new synthetic instances in the neighborhood of existing samples. On the other hand, undersampling techniques remove the linked and redundant major class samples to bring balance. However, hybrid sampling incorporates both strategies to eliminate the imbalance in the dataset.

Oversampling, also known as upsampling, is a sampling technique that helps to balance a dataset by duplicating minor class examples. This procedure has the advantage of causing little or no data loss. This approach has the problem of causing overfitting and adding to the computational load. The two types of oversampling are ROS and informative oversampling. ROS is a technique for balancing the distribution of minor classes by randomly repeating minor class examples. The informative oversampling technique [28] synthesizes minor class samples depending on a predefined criterion. Several applications of oversampling have been deployed for various types of datasets in recent years. A linear SVM was used in conjunction with a few SMOTE variations to detect malware in [29]. The model synthesizes dangerous occurrences based on the signature from the standpoint of the nominal properties. The malicious traffic dataset is first clustered using a single-linkage hierarchical technique to enrich the malicious class dataset, and then, signatures produced by every harmful traffic cluster are used. The resulting balanced datasets are then applied to train a semantic malware detection model for mobile devices. Random forest was used as the classifier with SMOTE to overcome the massive data imbalance problem. With a constrained hyperparameter set and nondynamic oversampling rate, SMOTE is used to eliminate imbalance after binarizing the original dataset [30]. This work fails for multiclass scenarios. In [31, 32], the same combination was used to detect insurance fraud claims and predict depression in women due to the modern lifestyle. B-SMOTE and SVM with kernel sigmoid were employed for data augmentation on P300 users with poor BCI performance [33]. For the DEAP dataset, the 1D-CNN model was utilized for classifications of two emotional dimensions: valence and arousal. B-SMOTE was employed to acquire a more homogeneous set of features of EEG signals [34]. For classifying HSIs, rotation forest has been combined with dynamic SMOTE [35], where SMOTE is applied to the imbalanced classes before each rotation tree is constructed. The procedure was discovered to take a long time. This work is expanded upon in [36], where the SMOTE technique is employed to create balanced datasets by incorporating spatial information from surrounding pixels of samples. These datasets are loaded into the weighted rotation forest model, which combines the RoF and multilevel cascaded RF. The cascade forest receives the rotation feature vectors generated by the rotation forest. In addition, the output likelihood of every level and the original data forms a stack. Furthermore, the sample weights need to be adjusted on a regular basis using the dynamic weight function generated from the classification scores at each level. According to [37], the adaptive synthetic sampling is another excellent strategy for oversampling when combined with a convolutional neural network to detect intrusion in a wireless network. ADASYN prevents the model from being sensitive to large samples but insensitive to small samples, which can help in small sample recognition and learning. The model considerably improves multiclassification jobs. However, a simpler residual network is still needed to increase small sample identification accuracy and execution performance. Another attempt at intrusion detection is made in [38], where ADASYN is applied to oversample the training dataset to enhance the number of infiltration and heartbleed attack behavior data samples. Classification and regression trees (CARTs) were used to create the DTs for the RF approach with a Gini coefficient. Even though this method delivers more incredible prediction performance, efficacy, and resilience, the parameters of ADASYN were adjusted artificially. All the above works that include oversampling suffer from a replicated voluminous data problem that is sometimes redundant, which leads to a common issue of high storage, calculation, and time complexity.

Undersampling, also known as downsampling, is a proper data balancing technique. The classifier obtains training using a major class subset in this method. When we undersample, some samples are deleted from the major class. Random sampling and informative undersampling are the two types of undersampling algorithms. The principle of random undersampling is simple: samples from the major class are randomly removed until the dataset is balanced. The informative undersampling technique selects only the necessary major class instances based on a prespecified selection criterion to balance the dataset [28]. Random undersampling [39] has the most relevance in huge data settings since it aids the random forest in making more accurate classifications in less time. In [40], the same methodologies are used for extensive specialized data for bioinformatics, where feature selection (FS) is used in conjunction with RUS, and the relationship between the two is investigated. The random forest learner is used in the FS component to compute feature importance, and encoding is used to transform categorical features into duplicate variables. RUS has a speedier runtime and a lower computing burden than random oversampling; however, the classification technique requires to be appropriate. Tomek connections have been demonstrated in [41], where TLs are used to eliminate outliers, noisy, and redundant samples from the major class of 10 real-world datasets. The removal of potentially ineffective examples causes the decision boundary to shift towards the minority region, providing a favorable environment for learning on various classifiers, including SVM. To create an application-oriented multiclass real-life application, the model must be supplemented with multiple schemes/techniques to eliminate the majority of instances with minimal data loss and faster processing. For the overpopulated bacterial data, in the preparation phase, the TL algorithm is used to clean data and reduce noise and produce a better result than oversamplers [42]. In addition, Tomek linkages are utilized to correct the imbalance in some medical datasets [43], where balanced data are put into the stacking ensemble after downsampling. It works on two levels. At level 0, there are many different classifiers, such as NB and SVM. The level 0 output is given to the level classifier for the final forecast. Base classifiers such as LR, k-NN, and NB are applied to apply datasets that are not more accurate and specific than contemporary research. The works discussed above used limited datasets and basic classifiers. On the one hand, undersampling might reduce the computational burden, but on the other hand, it may also remove significant information that might produce a better outcome.

Hybrid sampling is an appropriate combination of oversampling and undersampling procedures that correctly balance the training data. The oversampling strategy is used to create fresh samples by randomly sampling the current training data with replacement. Because the minor class is oversampled, a new balanced training dataset is created. The undersampling method is then used to reduce unwanted overlap between classes, lowering the number of classes. As a result, until a more assertive threshold for classifier conclusions could be created, the majority of data was eliminated [26]. Despite the small numbers, there is a significant study in this sector. Intrusion detection is carried out in [44] utilizing a mix of synthetic minority sampling and a neighborhood cleaning rule. For the learning process to be unaffected by data distribution, SMOTE generates a small number of datasets. Furthermore, the explored dataset revealed that border and noisy data have an impact on classification performance. As a result, NCL rules remove noisy and boundary data from the oversampled data. C4.5 and SVM are used as classifiers, but the model is not robust. The same hybrid resampling technique is used in conjunction with logistic regression [45]. This model is proven to be the most effective for binary categorized datasets. Another hybrid strategy combines synthetic oversampling with Tomek linkages, which have been used to detect fake credit cards [47] and medical disease datasets [46]. Overall, hybrid techniques are more prone to data loss and consume additional time.

Both works are based on a comparison of results from various classifiers. The student sadness data are distributed across universities using a combination of random oversampling, Tomek connections, and random forest. Only binary and less noisy datasets are adequate for this model [48]. A hybrid of SMOTE and ENN is used to process the KDDCup99 dataset and tackle the difficulties of data imbalance and sample overlapping with the classifier RF [49]. The same combination but with classifier XGBoost is carried forward to build a prediction model that efficiently determines the category of a person, whether healthy or possessing Parkinson's syndrome [50]. RRE pruning is used for HSI classification that prunes the constituent classifiers with poor complementarity, and subsequently, the leftover constituent classifiers with higher complementarity are joined to produce an ensemble classifier. These strategies ensure that the component classifiers used to build the ensemble classifier are precise but diversified, which enhances the ensemble classifier's performance [51].

## 3. Methodology

**Figure 2:**
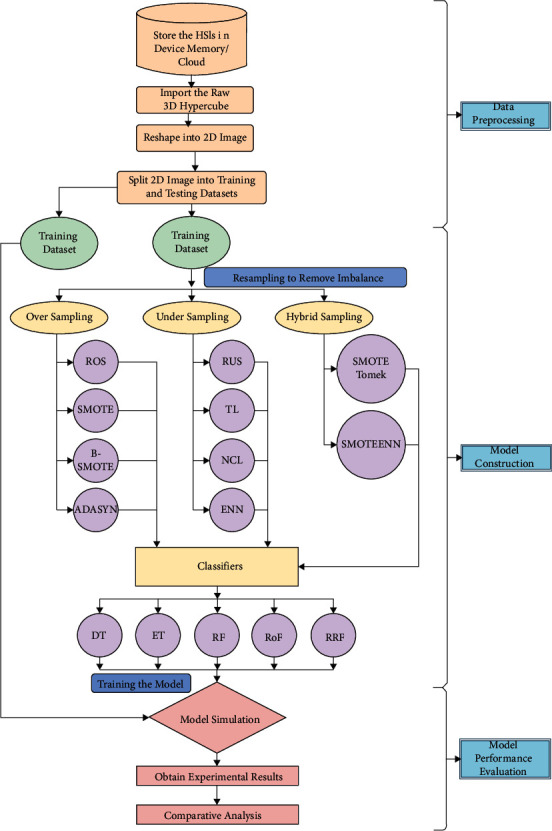
Workflow of the proposed model.

Figure 2 displays the framework of our suggested approach for improving hyperspectral image categorization by coping with sample imbalance. The following are the steps that are included in structuring our study.

### 3.1. Data Preprocessing

#### 3.1.1. Dataset

**Figure 3:**
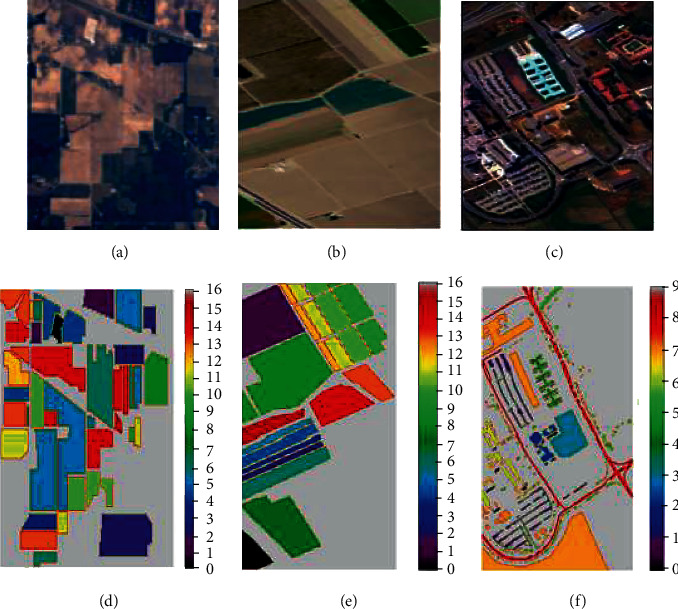
Composite color images (CCIs) of the hyperspectral datasets such as (a) IP, (b) SV, (c) PU and the ground truth images (GTIs), (d) IP, (e) SV, and (f) PU.

For our experiment, we collected three mostly explored hyperspectral datasets that are available in the public domain [52] and stored in the memory of our system. A brief elaboration of the datasets is as follows, along with Figure 3.

- *AVIRIS Indian Pines* (*IP*): this dataset was captured by Airborne Visible/Infrared Imaging Spectrometer (AVIRIS) sensor on June 12, 1992. The scene captured here was the Indian Pines test site in northwestern Indiana, USA. It contains an agricultural area exemplified by its crops of common geometry and some irregular forest zones. It consists of 145 *∗* 145 pixels with a spectral resolution of 10 nm and spatial resolution of 20mpp and 224 spectral reflectance bands in the wavelength range of 0.4–2.5 *μ*m, of which 24 noisy bans are removed due to low signal-to-noise ratio. In addition, the scene contains 16 different classes of land covers. The IP dataset acquires ImbR = 73.6.
- *Salinas Valley* (*SV*): this scene was obtained by AVIRIS sensor over various agricultural fields of Salinas Valley, California, USA, in 1998. The scene is characterized by a high spatial resolution of 3.7mpp and spectral resolution of 10 nm. The area is covered by 512 *∗* 217 spectral samples with a wavelength range of 0.4–2.5 *μ*m. Of 224 reflector bands, 20 noisy bands are discarded due to water absorption coverage. The scene comprises 16 different land classes. The SV dataset acquires ImbR = 12.51.
- *Pavia University* (*PU*): this scene was captured by a Reflective Optics System Imaging Spectrometer (ROSIS-03) sensor during a flight campaign over the University of Pavia in 2001. It possesses 115 spectral bands, of which only 103 are useable. Its spectral coverage is 0.43–0.86 *μ*m, with spectral resolution of 4 nm and spatial resolution of 1.3mpp defined by 610 *∗* 340 pixels. The scene contains 9 classes with urban environmental constructions. The PU dataset acquires ImbR = 19.83.

#### 3.1.2. Loading the Dataset and Splitting

The datasets are imported as hypercubes and converted into a processable three-dimensional format. Then, the 3D images are reshaped into a machine-readable 2D format. The dataset is further broken up into training and testing datasets in a ratio of 3 : 2; i.e., we have used 60% of the original individual datasets for training our model, and the residual 40% is set aside for testing the model's performance. The training set is processed from the next step onwards, while the testing dataset remains intact. The training and testing samples for each dataset are depicted in Table 1.

### 3.2. Data Balancing by Resampling

#### 3.2.1. Oversampling Techniques

*(1) Random Oversampling (ROS)*. ROS [18] involves selecting random examples from the minority class with replacements and augmenting the training data with numerous copies of the particular instances so that a specific instance could be chosen many times. Overfitting has been shown to be more likely when ROS is applied.

*(2) Synthetic Minority Oversampling Technique (SMOTE)*. Chawla et al. [19] presented SMOTE as an oversampling strategy to avoid the overfitting problem. This method is considered cutting edge and is effective in a wide range of applications, including the HSIs. This approach generates synthetic data based on feature space resemblances between prevailing minority occurrences. Making an artificial instance determines each minority instance's k-NN, chooses one at random, and then uses linear interpolation to create a new minor instance in the neighborhood. The detailed algorithm of SMOTE is as follows:

Step 1: *k*-nearest neighbors are calculated with minor class samples following Euclidean distance for each minority instance *x*_i_.
Step 2: a neighbor *x*_*j*_ is picked in a random manner from the *k*-nearest neighbors of *x*_*i*_.
Step 3: new samples *x*_new_ are produced in between *x*_*j*_ and *x*_*i*_:
  (2) $$\begin{array}{c} x_{\text{new}}=x_{i}+\;\beta \left|x_{i}\;–\;x_{j}\right|, \end{array}$$
where *β* is the random number between 0 and 1.

*(3) Borderline SMOTE (B-SMOTE)*. B-SMOTE [20] creates a synthetic sample dividing minor and major groups. This method also aids in the division of the minor and major groups. The minor class observations are first classified using this approach. If all of the neighbors are in the major class, it identifies any minor observation as noise and ignores it while synthesizing synthetic data. Furthermore, it resamples completely from a few border locations that include major and minor classes as neighborhoods.

*(4) Adaptive Synthetic Minority Oversampling Technique (ADASYN)*. Haibo He et al. [21], inspired by SMOTE, present ADASYN technique, which has received considerable attention. ADASYN generates minor class samples based on their density distributions. Compared with minority class samples that are simpler to learn, more artificial data are produced for minor class samples that are challenging to learn. It computes each minor instance's k-NN and then uses the class ratio of the minor and major examples to produce fresh samples. It adaptively alters the decision boundary to concentrate on those samples that are challenging to learn by repeating this process. ADASYN enhances learning of data distribution in two ways: (1) minimizing the bias created by the class imbalance and (2) adjusting the classification decision boundary in the direction of the complicated examples.

Training dataset DTR is presumed with *n* samples {*x*_*i*_, *y*_*i*_}, *j* = 1,…, *n*, where *x*_*i*_ is an example belonging to the *n*-dimensional feature space *X* and *y*_*j*_ ∈ *Y* = {1, −1} defines the class label coupled with *x*_*j*_, and *n*_*s*_ and *n*_*l*_ denote the number of minority and major class examples, respectively. Thus, *n*_*s*_ ≤ *n*_*l*_ and *n*_*s*_ + *n*_*l*_ = *n*. Based on these notations, the following steps are to be followed:

Step 1: compute the degree of class imbalance: *i* = *n*_*s*_/*n*_*l*_, range of *i* ∈ (0, 1].
Step 2: compute the quantity of synthetic data samples that the minor class needs to produce:
  (3) $$\begin{array}{c} R=\left(n_{l}-n_{s}\right)\times \beta , \end{array}$$
where *β* ∈ [0, 1] is the constraint for specifying the required balance level after the synthetic data creation.
Step 3: for every sample *x*_*j*_ ∈ minor class, find *k*-nearest neighbors found on the basis of Euclidean distance in *n*-dimensional space, and compute the ratio *r*_*i*_ described as follows:
  (4) $$\begin{array}{c} P_{j}=\frac{\Delta_{i}}{k},\;j=1,\ldots ,n_{s}, \end{array}$$
where Δ_*i*_ denotes the amount of samples in the *k*-nearest neighbors of *x*_*i*_ that belong to the major class; thus, *P*_*j*_ ∈ [0, 1].
Step 4: standardize *P*_*j*_ according to ${\hat{P}}_{j}=P_{j}/\sum_{j=1}^{n_{a}}P_{j}$ so that ${\hat{P}}_{j}$ denotes a density distribution $\left(\sum_{j}{\hat{P}}_{j}=1\right)$.
Step 5: calculate the exact number of synthetic data samples that need to be produced for every minority sample *x*_*j*_:
  (5) $$\begin{array}{c} r_{i}={\hat{P}}_{j}\times R, \end{array}$$
where *R* is the overall figure of synthetic data instances that needs to be produced for the minor class.
Step 6: for every minor class data instance *x*_*i*_, produce *r*_*i*_ synthetic data samples by choosing one minor data example in a random way, *x*_*zj*_, from the *k*-nearest neighbors for data *x*_*j*_:
  (6) $$\begin{array}{c} s_{i}=x_{j}+\left(x_{zj}–x_{j}\right)\times \alpha , \end{array}$$
where (*x*_*zj*_ − *x*_*j*_) is the contrast vector in the *n*-dimensional space, and *α* is an arbitrary number: *λ* ∈ [0, 1].

#### 3.2.2. Undersampling Techniques

*(1) Random Undersampling (RUS)*. The inverse of ROS is RUS [22]. This approach aims to choose and eliminate samples from the major class at random, diminishing the number of examples in the modified data from the major class. However, RUS has the significant disadvantage of discarding useful information.

*(2) Tomek Links (TLs)*. TL is a variant of Tomek's condensed nearest neighbor (CNN) undersampling algorithm [23]. Unlike the CNN technique, which selects samples with their k-NNs from the major class that has to be deleted at random, the TL method employs a rule to select pairs of observations (suppose A and B) that meet the following criteria: (1) the observation B is A's closest neighbor; (2) the observation A is B's closest neighbor; and (3) observations A and B are from distinct classes; i.e., A and B are members of the minor and major classes, respectively, or vice versa.

*(3) Neighborhood Cleaning Rule (NCL)*. NCL [24] is an undersampling strategy that reduces data based on cleaning to overcome the imbalanced class distribution. One of the benefits of NCL is that it examines the data quality to be destroyed rather than focusing solely on the reduction in data. The data cleansing procedure is intended for samples from major and minor classes. Essentially, NCL is built on the notion of one-sided selection (OSS), a technique to reduce data based on incidences to decrease classes carefully. On NCL, the cleaning data process is conducted independently of the major and minor samples.

*(4) Edited Nearest Neighbor (ENN)*. The ENN approach, which was developed by Wilson [25], works by first determining the *k*-NN of every observation and then determining whether the major class from the observation's *k*-NN is the same as the observational class or not. If the observation's *k*-NN's major class differs from the observation's class, the observation and its *k*-NN are removed from the dataset. This method is more potent than TL because ENN removes the observation and its *k*-NN when the observation's class and the major class from the observation's k-NN are different, rather than simply the observation and its 1-nearest neighbor. As a result, ENN is likely to provide more thorough data cleaning than TL.

#### 3.2.3. Hybrid Sampling Techniques

*(1) SMOTETomek*. This method, first proposed by Batista et al. [26], blends the SMOTE's ability to create synthetic data for the minor class with the TL's ability to eradicate data from the major class that is identified as TL, i.e., samples of data from the major class that is nearest to the minor class data.

*(2) SMOTEENN*. This method, established by Batista et al. [27], merges the ability of SMOTE to generate synthetic examples for minor classes with the ability of ENN to delete some observations from both classes. Those observations are identified as having different classes between the observation's class and its *k*-NN major class.

### 3.3. Tree-Based Ensemble Classifiers

*(1) Decision Tree (DT)*. The most widely used supervised data mining approaches is the DT algorithm [12]. DT uses a divide-and-conquer approach. The operating method is to find a feature possessing the best ability to classify and split data into many subsets in a recursive manner until a stopping criterion is fulfilled. The class is predicted using decision rules derived from the data input. Determining attribute selection parameters such as information gain or Gini index, the root represents the best feature. It can work with both numerical and categorical data. Furthermore, outliers and the missing values have a negligible impact on the model's results. However, DT uses a greedy technique, which might lead to overfitting [53]. The DT algorithm can be applied as a feature selection strategy in addition to a classification method [54]. The features used to construct splitting rules at internal tree nodes are DT feature selection results. DT is a filter strategy because it measures features rather than classification accuracy.

*(2) Random Forest*. (*RF*) RF is a well-known ensemble ML approach that stems from DT [13]. While building a model, it can manage the overfitting branch of DT. As a result, many classification models are created, each constructed using a feature selector such as the information gain, Gini index, and gain ratio.

These models realize and create an impact on the prediction in a discrete manner [53]. Random sample selection and random feature selection are the essential concepts. All trees in RF are independent of one another, allowing for parallel training and testing. Consider the dataset *S*_*n*_, which contains *n* samples (*U*, *V*), with *U* ∈ *R*^*S*^. To begin with, *m* instances are randomly chosen with replacements from the original dataset *S*_*n*_. The current decision tree is built using these examples. Second, from the initial *S* features, *p* features (*p* < *S*) are picked at random. CARTs are produced using the Gini impurity or mean-squared error criterion. Finally, using the majority vote criterion, the categorization result is produced [36].

*(3) Extra Trees*. (*ET*) ET [15] is an ensemble learning operating mechanism like RF. ET creates classification and regression by combining the results of a large number of uncorrelated trees. The first of two key differences between ET and RF is that ET samples do not require replenishment. The second is that it chooses random attributes to split the tree nodes rather than the best [53]. Furthermore, ET is preferable to RF in the sense that it is faster and allows for very little noise data.

*(4) Rotation Forest*. (*RoF*) Rodriguez proposed a rotation forest in 2006 [16], based on the random forest concept. Feature transformation being the basic idea behind this algorithm, it aims to enhance the difference and accuracy of the underlying classifier. The following steps are used to create a T-size rotation forest model.

  Step 1: The feature space denoted as *F* is segregated into *N* disjoint sets of features, and every subset contains features of *K* (=*F*/*N*) number.
  Step 2: A new train set is attained by utilizing a bootstrap algorithm to select 75% of the training data randomly.
  Step 3: the coefficient *A*_*v*,*h*_ (*h* ≤ *H*, *v* ≤ V) is achieved using the principal component analysis on every sub-feature space *F*_*v*,*h*_ (*h* ≤ *H*, *v* ≤ V), and the coefficients belonging to each subspace are arranged in a sparse rotation matrix *R*_*v*_ (*v* ≤ V).
  Step 4: The columns of *R*_*v*_ are rearranged by duplicating the order of initial features *F* to produce the rotation matrix *R*_*v*_′. The new train set *B*_*t*_′ = [*B*_*v*_*R*_*v*_′, *Y*_*t*_] is produced for training a specific classifier.
  Step 5: The process mentioned above is repeated on all different train sets and a sequence of specific classifiers is produced. The majority voting rule achieves the final result.

*(5) Random Rotation Ensemble Forest*. (*RREF*) The idea of RRE was proposed by Blaser and Fryzlewicz [17] in 2016. Regardless of utilizing the identical sequence of the random numbers in the RF algorithm's tree induction phase, similar bootstrap samples and associated feature subset selections at every decision branch for both the trees are made. The random feature rotation has a considerable influence on the resultant data partition. The subsequent tree is not simply a rotated form of the unrotated tree; it has an entirely different orientation and data division. Samples are consistently distributed throughout all possible rotations to execute a random rotation. For *n* > 2, where *n* denotes the number of independent normal variates, rotating every angle in spherical coordinates at random does not result in a consistent distribution over all the rotations, implying that some rotations are more common than others.

Let us consider *x* as the unit vector directing towards the *n*-spherical space at an arbitrary point. The classification trees *T* split the predictor space into *D*_*i*_ disjoint regions, with 1 ≤ *i* ≤ *I*, where *I* denotes the total count of the terminal nodes of *T*. The random and optimization parameters are denoted by *α* = {*G*, *w*} and *β* = {*D*_*i*_, *v*_*i*_}_1_^*I*^, respectively, where *G* is the random rotation coupled with *T*, w is the arbitrary tree induction sample pairs, and every randomly rotated input *G* (*x*) performs a mapping to a constant *v*_*i*_, which depends on the belonging of the input to a particular region of *D*_*i*_. Thus, the tree with an indicator function *J* (.) formulates as follows:

(7) $$\begin{array}{c} T\left(x;\alpha ,\beta \right)=\sum_{i=1}^{I}v_{i}J\left(G\left(x\right)\in D_{i}\right). \end{array}$$

### 3.4. Model Simulation

A classification model is built using each of the 10 resampling techniques taken one at a time. The balanced data are then classified using each of the 5 tree-based ensemble classifiers separately, as shown in Figure 2. Therefore, our comparative experiments consist of a total of 50 training models for each dataset. Every model construction requires a suitable hyperparameter setting. For oversampling, we have mostly chosen the minor classes to create a similar number of samples as the major classes; thus, the sampling strategy is “minority.” Correspondingly, the sampling strategy for undersampling techniques is chosen “majority,” where the redundant major class samples are removed to match up with the minor class samples. We first set the base oversampler and undersampler with previously stated parameters and then set the sampling strategy as “all” to the hybrid sampler for the hybrid sampling. Also, for SMOTE and ADASYN, we have taken 5 *k*-nearest neighbors, whereas for NCL and ENN we chose 3 *k*-nearest neighbors. The hyperparameters that are set for the classifiers are shown in Table 2.

## 4. Experimental Outcomes

### 4.1. Experimental Setup

All program codes are implemented using Python language with its latest versions of embedded packages, such as Keras, TensorFlow, and scikit. The hardware specifications are Intel® Core™ i5-10300H Processor, 2.5 GHz, 8 GB DDR4 2933 MHz RAM, and 4 GB NVIDIA GeForce GTX 1650 Ti. After splitting the original dataset into train and test sets, we (1) apply the resampling techniques, viz. 4 oversampling, 4 undersampling, and 2 hybrid sampling strategies to our datasets individually; (2) train each of the tree-based ensemble classifier models with each resampling method; (3) obtain the classification performance using the assessment metrics; and (4) present a detailed comparative analysis based on the obtained metric statistics. We have uniformly overpopulated the selected minor class samples and removed neighborly linked major class samples to balance the individual dataset. The same strategy is used in combination for the hybrid sampling. The hyperparameter setting for the classifiers is as follows:

1. For DT, ET, RF, and RREF, we have used the Gini criterion with a maximum tree depth of 100 and the number of estimators as 1000, keeping the other hyperparameters as default.
2. For RoF, we have taken 1000 number of trees and a total of 20 features, keeping the rest as default.

### 4.2. Performance Evaluation Metrics (PEM)

In this work, we have adopted five prime metrics to assess our model's performance: precision, recall, *F* score, Cohen kappa, overall accuracy, and the time elapsed to execute the entire process. These are described as follows.

Let us denote *Y*=the total number of class labels in the dataset, bii = true prediction of ith class, *b*_*ji*_ = false prediction of *i*th class, and *b*_*ij*_ = false prediction of *i*th class into *j*th class.

*(1) Precision Score.* Precision is used to assess each class classification accuracy in the imbalanced data. The precision score is expected to be high for a better classifier. The precision score (Prec_score%) measures the testing prediction rate of all samples and is defined in the following equation:

(8) $$\begin{array}{c} \text{Prec}\_\text{Score}\%=\frac{1}{Y}\frac{b_{ii}}{\sum_{i=1}^{Y}b_{ji}}\times 100. \end{array}$$

*(2) Recall Score.* Recall or true-positive rate is the percentage of correctly classified events. The recall is especially well suited to assessing classification systems dealing with many skewed data classes. The large recall value indicates the better performance of a classifier. The Rec_score% is given by the following equation:

(9) $$\begin{array}{c} \text{Rec}\_\text{Score}\%=\frac{1}{Y}\sum_{i=1}^{Y}\frac{b_{ii}}{\sum_{i=1}^{Y}b_{ij}}\times 100. \end{array}$$

*(3) F_Score.* In the classification of imbalanced data, the *F*-measure, an assessment index derived by combining precision and recall, has been widely employed. The introduction of *F*-measure combines the two, and the greater the *F*-measure, the better the classifier's performance. The computation of *F*_score% is given as follows:

(10) $$\begin{array}{c} F\_\text{Score}\%=\frac{2}{Y}\frac{\sum_{i=1}^{Y}R_{i}\cdot \sum_{i=1}^{Y}P_{i}}{\sum_{i=1}^{Y}R_{i}+\sum_{i=1}^{Y}P_{i}}\times 100, \end{array}$$

where *R*_*i*_ and *P*_*i*_ denote the precision and recall of class *i*, respectively.

*(4) Cohen_Kappa_Score.* Cohen kappa is a statistic that evaluates the predictability of the findings and determines whether the consistency is genuinely random. The greater the Cohen_kappa, the better the classifier's performance. Kappa_score% is equated as follows:

(11) $$\begin{array}{c} \text{Kappa}\_\text{Score}\%=\frac{\text{Overall Accuracy}-\sum_{i=1}^{Y}q_{i}\cdot q_{i}^{\prime }}{1-\sum_{i=1}^{Y}q_{i}\cdot q_{i}^{\prime }}\times 100, \end{array}$$

where *q*_*i*_ and *q*_*i*_′ denote the original and predicted sample sizes of class *i*, respectively.

*(5) Overall Accuracy.* Overall accuracy, being a performance-metric, assigns the similar weight to every of the data types, regardless of their number of instances. The definition of OA is given as follows:

(12) $$\begin{array}{c} OA\%=\frac{1}{Y}\sum_{i=1}^{Y}\frac{b_{ii}}{\sum_{i=1}^{Y}\left(b_{ji}+b_{ii}\right)}\times 100. \end{array}$$

*(6) Time Elapsed.* Time complexity is a factor in real-time computation. Lesser the time complexity, the higher the quality of the classifier. Time elapsed for execution is defined as follows:

(13) $$\begin{array}{c} TE\left(\text{in seconds}\right)=\text{End time of execution}–\text{Start time of execution.} \end{array}$$

### 4.3. Comparative Performance Analysis of the Model

#### 4.3.1. For Indian Pines Dataset

*(1) Effect of Oversampling*.  Table 3 describes the comparison between the tree-based classifier model performances due to the oversampling of the data. It is evident that SMOTE achieves better results in all performance metrics for all classifiers. RREF attains the highest accuracy of 89.49%, with an approximation of 1.32, slightly higher than RoF. Also, the total time consumed is maximum for ADASYN for all the classifiers, especially for RoF.

*(2) Effect of Undersampling*.  Table 4 describes the comparison between the tree-based classifier model performances due to the oversampling of the data. It is imperative that TL achieves better results in all performance metrics for all classifiers, except for DT, where NCL attains the highest OA. RREF attains the highest accuracy of 85.32%, with an approximation of 2.76, which is slightly higher than RoF. Also, the total time consumed is maximum for TL for all the classifiers, especially for RoF.

*(3) Effect of Hybrid Sampling*.  Table 5 compares the tree-based classifier model performances due to the hybrid sampling of the data. It can be inferred that SMOTETomek achieves better results in all performance metrics for all classifiers. RoF attains the highest accuracy of 82.74%, with an approximation of 2.16, slightly higher than RoF. Also, the total time consumed is maximum for SMOTETomek for all the classifiers, especially for RoF.

#### 4.3.2. For Salinas Valley Dataset

*(1) Effect of Oversampling*.  Table 6 compares the tree-based classifier model performances due to the oversampling of the data. ADASYN achieves better results in all performance metrics for all classifiers. RREF attains the highest accuracy of 95.84%, with an approximation of 1.33, slightly higher than RoF. Also, the total time consumed is maximum for ADASYN for all the classifiers, especially for RoF.

*(2) Effect of Undersampling*.  Table 7 describes the comparison between the tree-based classifier model performances due to the oversampling of the data. It is imperative that TL achieves better results in all performance metrics for all classifiers. RoF attains the highest accuracy of 95.41%, with an approximation of 1.5, which is slightly higher than RREF. Also, the total time consumed is maximum for TL for all the classifiers, especially for RoF.

*(3) Effect of Hybrid Sampling*.  Table 8 describes the comparison between the tree-based classifier model performances due to the hybrid sampling of the data. It can be inferred that SMOTETomek achieves better results in all performance metrics for all classifiers. RoF attains the highest accuracy of 94.89%, with an approximation of 2.24, which is slightly higher than RoF. Also, the total time consumed is maximum for SMOTETomek for all the classifiers, especially for RoF.

#### 4.3.3. For Pavia University Dataset

*(1) Effect of Oversampling*.  Table 9 depicts the comparison between the tree-based classifier model performances due to the oversampling of the data. It is evident that SMOTE achieves better results in all performance metrics for all classifiers. RoF attains the highest accuracy of 91.89%, with an approximation of 1.45, which is slightly higher than RoF. Also, the total time consumed is maximum for ADASYN for all the classifiers, especially for RoF.

*(2) Effect of Undersampling*.  Table 10 describes the comparison between the tree-based classifier model performances due to the oversampling of the data. It is imperative that TL achieves better results in all performance metrics for all classifiers, except for DT, where ENN attains the highest OA. RoF attains the highest accuracy of 89.91%, with an approximation of 1.34, which is slightly higher than RREF. Also, the total time consumed is maximum for TL for all the classifiers, especially for RoF.

*(3) Effect of Hybrid Sampling*.  Table 11 describes the comparison between the tree-based classifier model performances due to the hybrid sampling of the data. It can be inferred that SMOTETomek achieves better results in all performance metrics for all classifiers. RREF attains the highest accuracy of 85.83%, with an approximation of 2.32, which is slightly higher than RoF. Also, the total time consumed is maximum for SMOTETomek for all the classifiers, especially for RoF.

#### 4.3.4. Comprehensive Discussion

**Figure 4:**
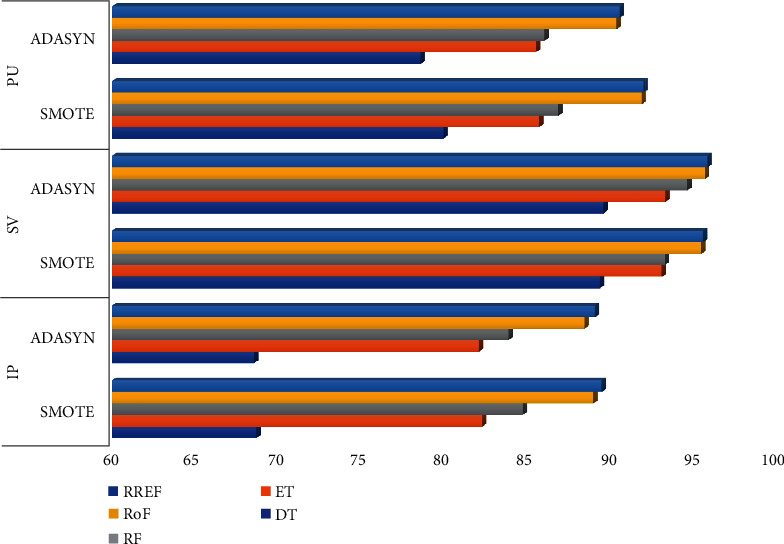
OA% comparison between SMOTE and ADASYN associated with the tree-based ensemble classifiers for IP, SV, and PU datasets.

**Figure 5:**
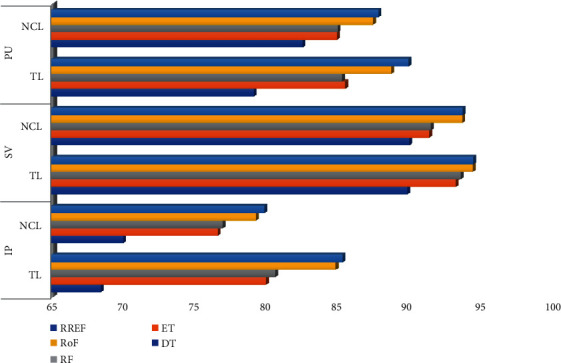
OA% comparison between TL and NCL associated with the tree-based ensemble classifiers for IP, SV, and PU datasets.

The total number of land cover pixels in each band of the IP, SV, and PU datasets is 10249, 54129, and 42776, respectively. Also, SV represents a valley scene, whereas the others represent urban sites. From the tables above, certain inferences can be drawn. As an oversampling technique, SMOTE stands out to be best for IP and PU datasets, but for SV, ADASYN produces the best result throughout all classifiers. SMOTE and ADASYN achieve better outcomes for all the datasets than other oversampling methods.  Figure 4 depicts the graphical comparison of the performances of SMOTE and ADASYN in terms of OA%. TL is the best technique for achieving good results for the HS datasets for the undersampling approach. The statistics for NCL have been closer to the outcomes of TL, although it has outperformed TL with DT classifier for all the datasets.  Figure 5 represents the performance comparison between TL and NCL based on OA%. SMOTETomek has surpassed SMOTEENN in all aspects of performance for all the datasets.

**Figure 6:**
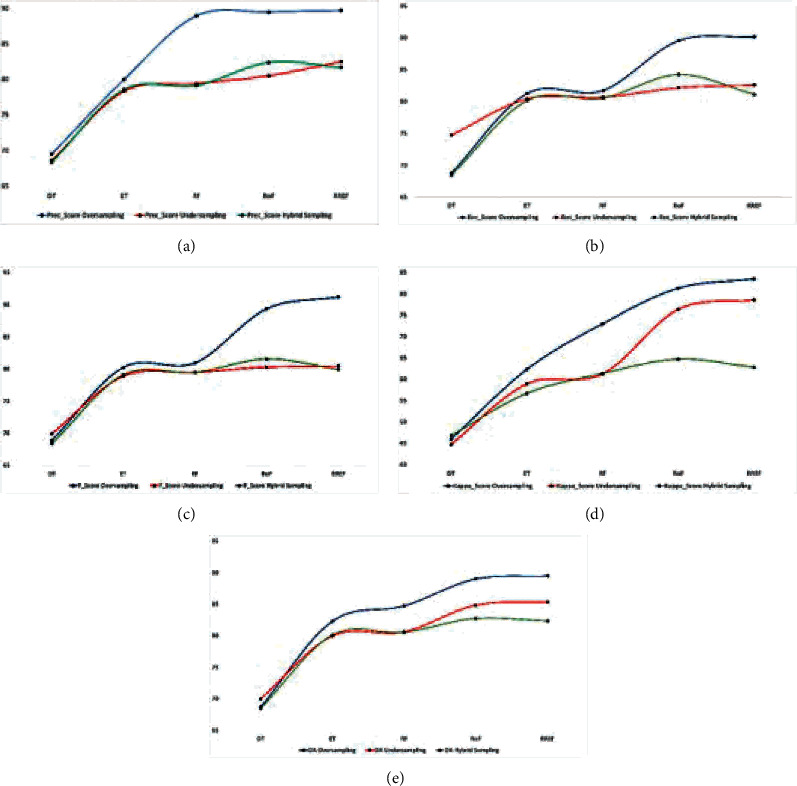
Comparison of the PEM for oversampling, undersampling, and hybrid sampling applied on the IP dataset. (a) Prec_score%. (b) Rec_score%. (c) *F*_score%. (d) Kappa_score%. (e) OA%.

**Figure 7:**
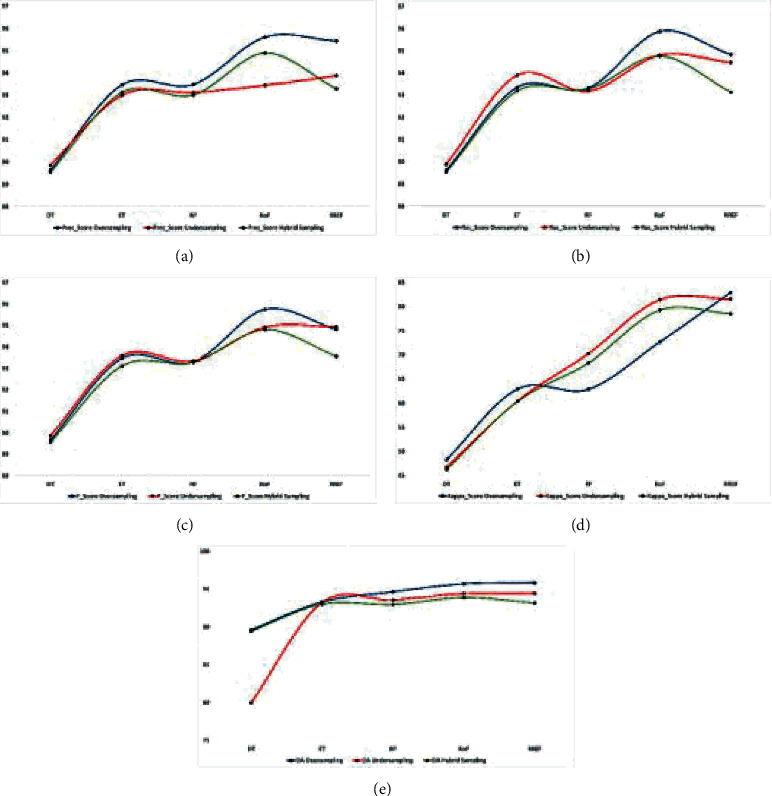
Comparison of the PEM for oversampling, undersampling, and hybrid sampling applied on the SV dataset. (a) Prec_score%. (b) Rec_score%. (c) *F*_score%. (d) Kappa_score%. (e) OA%.

**Figure 8:**
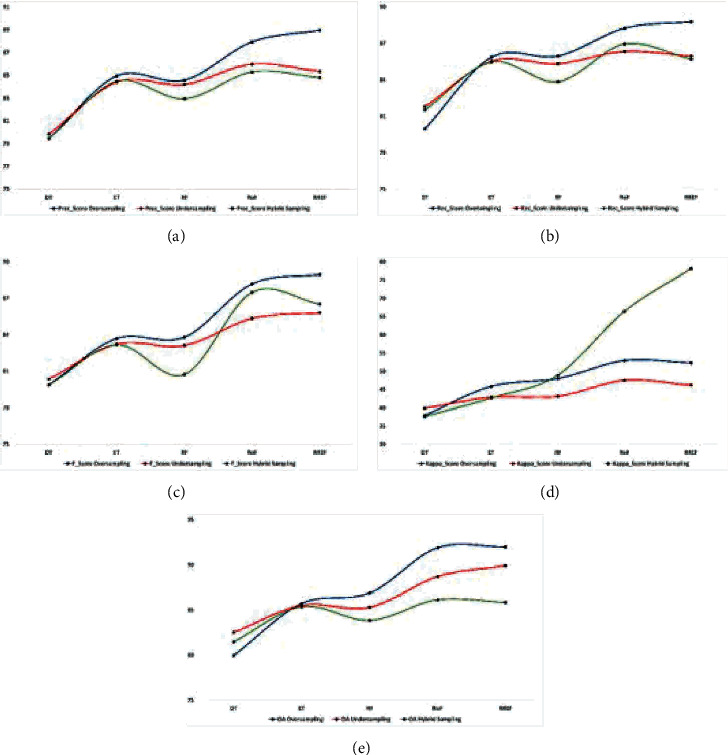
Comparison of the PEM for oversampling, undersampling, and hybrid sampling applied on the PU dataset: (a) Prec_score%. (b) Rec_score%. (c) *F*_score%. (d) Kappa_score%. (e) OA%.

Figures 6–8 represent all EM data plotted in graphs to understand better the effects of resampling techniques on the HS datasets, viz. IP, SV, and PU. The blue, red, and green curves represent the oversampling, undersampling, and hybrid sampling technique that produces a better result than other resampling techniques. We can infer the more appropriate strategy for further research from these graphical illustrations. Figures 6(a)–6(e) show that the PEMs, viz. precision, recall, *F*_score, kappa, and OA of the oversampling techniques with the classifier RREF, are the highest for the IP dataset, whereas DT is the least impactful. For hybrid sampling, RoF achieves the best overall PEM scores. The SV dataset obtains the best outcome in oversampling and RREF, whereas there is only a little difference between the performance measures, statistically, that dwells between RoF and RREF. The same scenario applies to the PU dataset in terms of PEMs, as shown in Figures 7(a)–7(e). For the IP dataset, represented by Figures 8(a)–8(e), with the lowest dimension containing less spectral resolutions, RREF performs best for oversampling and undersampling, but RoF stands out in hybrid sampling. The other two datasets, SV and PU, have higher dimensions enriched with more differentiative spectral features. When they undergo undersampling or hybrid sampling, RoF makes the most corrective decisions. These figures conclude that DT, a simple and basic tree-based ensemble classifier, produces the least accuracy for all datasets. However, RREF is an ensemble of two efficient and state-of-art tree-based ensemble classifiers. It generates maximum OA for all datasets associated with the oversampling techniques, SMOTE and ADASYN, and the undersampling technique, TL. However, hybrid sampling is found to be more compatible with RoF.

**Figure 9:**
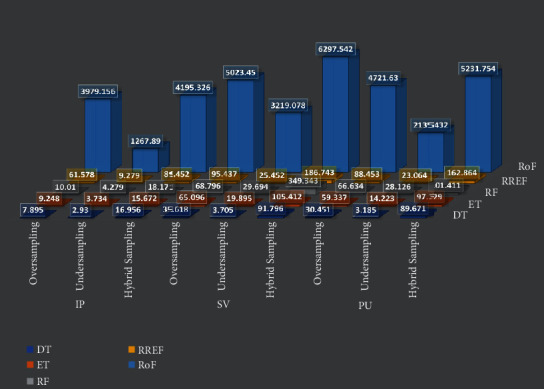
Comparison of maximum TE (in secs) for the oversampling, undersampling, and hybrid sampling techniques taken for each of the classifiers applied on IP, SV, and PU datasets.

The calculation and comparison of elapsed time for executing the entire training system, i.e., the time complexity, also play a vital role. This comparison is graphically illustrated in Figure 9 as a 3D bar chart. The figure shows that hybrid sampling is the amalgamation of an oversampler, and an undersampler holds a more complex structure and takes the highest time to build the model. Then come the oversampling techniques that generate synthetic samples to recreate the balanced dataset, which takes a certain amount of time. Finally, the order is followed by undersampling strategies whose elapsed time is least due to the deletion of linked neighborhood data samples. This order is thoroughly followed irrespective of the type of the applied tree-based ensemble classifier.

The comparison between the elapsed time with units in seconds is also inevitable for the different ensemble tree-based classifiers we used in our work. The order of increment in time of execution for those classifier models is consistent: DT < ET < RF < RREF << RoF. According to the previous discussion, RREF and RoF provide the best outcomes for all the resampling strategies. The time taken by RoF is at most 136.85 times (undersampling in IP) and at least 32.12 times (hybrid sampling in PU) higher than RREF. The other TE (in secs) ratios for RREF and RoF, as shown in Figure 9, lie within the said range.

The entire study summarizes that oversampling strategies are more compatible with hyperspectral images as they are more consistent than undersampling and hybrid sampling strategies. Oversampling provides the creation of new synthetic instances that inevitably regenerate the existing dataset and bring in a balance between the samples of major and minor classes. Due to the overpopulation of the samples, the dataset does not suffer from the lack of labeled data, and no feature or information is lost, which is an issue in undersampling. The fully balanced dataset is then fed to the tree-based ensemble classifier models as input. The decision trees are made in equilibrium with the balanced samples and produce elementary decisions. Those decision outcomes are passed into forests, and the averaged classification result is ultimately obtained. However, from Tables 2, 5, and 8, we found that SMOTE is mostly better than ADASYN when the ratio of class imbalance is high. This is due to the SMOTE-augmented minority class overall anticipated value being the same as the original minority class expected value, but its variance is lower. As a result, SMOTE has little effect on classifiers that use mean values and total variances to determine categorization rules. ADASYN is also helpful in reducing the learning bias caused by the data distribution of the original imbalanced dataset. The disadvantage of ADASYN over SMOTE is that the procedure is more complex and time-consuming due to its adaptive nature. Additionally, the contrasts are found to be minor between the PEMs derived by RREF and RoF as tree-based ensemble classifier models with each of the oversampling, undersampling, and hybrid sampling procedures. Finally, we can say that the classifier RREF, in combination with the oversampling algorithms such as SMOTE and ADASYN, is capable of producing outstanding classification results for balanced hyperspectral datasets.

## 5. Conclusion

Data imbalance has been a delicate issue in big data scenarios with enormous and high-dimensional data. Due to imbalance, classifiers suffer from low accuracy and quality. In our work, we have offered a brilliant study of comparison between the effects of oversampling, undersampling, and hybrid sampling on three highly imbalanced hyperspectral datasets. Furthermore, we rely on the fact presented by previous researchers that the tree-based ensemble classifiers are more useful when the data samples belonging to different classes are nearly balanced. As an effect, we have incorporated a handful of eminent ensemble tree strategies that have achieved remarkable outcomes. For building our models for each resampling strategy and the individual classifier, we executed 50 models for each dataset. For all HS datasets, our findings revealed that in oversampling, SMOTE and ADASYN, while in undersampling, Tomek Links, and in hybrid sampling, SMOTETomek techniques are more compatible with RoF and RREF. Practically and experimentally, oversamplers achieve higher performance statistics than other resampling techniques as they sustain and sometimes add further features for better classification. On the other hand, in undersampling and hybrid sampling, there is a provision for removing redundant data from major classes, which sometimes may lead to loss of information, which affects the classification performance abruptly. Furthermore, there is enough scope to improve all the sampling strategies to become compatible with voluminous real-life datasets for robust applicabilities.

As a limitation to our present work, we can list specific points: (1) the classifiers used need to be cross-validated to produce more optimized outcomes; (2) a limited number of resampling techniques are used with limited hyperparameters; and (3) the computation and time complexity are high. In the future, we plan to incorporate recently developed variants of SMOTE and ADASYN along with more efficient forest ensembles. Also, we will try to explore the TL and its possibility of being optimized to achieve a more accurate outcome.

## Figures and Tables

**Table 1:** Original ground truth, training, and testing samples of IP, SV, and PU datasets.

The dataset	Indian Pines				Salinas Valley				Pavia University			
Class no.	Class name	Original	Train	Test	Class name	Original	Train	Test	Class name	Original	Train	Test
1	Alfalfa	46	28	18	Brocoli_green_weeds_1	2009	1205	804	Asphalt	6631	3979	2652
2	Corn-notill	1428	857	571	Brocoli_green_weeds_2	3726	2236	1490	Meadows	18649	11189	7460
3	Corn-mintill	830	498	332	cFallow	1976	1186	790	Gravel	2099	1260	839
4	Corn	237	142	95	cFallow_rough_plow	1394	836	558	Trees	3064	1838	1226
5	Grass-pasture	483	290	193	Fallow_smooth	2678	1607	1071	Painted metal sheets	1345	807	538
6	Corn-trees	730	438	292	Stubble	3959	2375	1584	Bare soil	5029	3018	2011
7	Corn-pasture-mowed	28	17	11	Celery	3579	2147	1432	Bitumen	1330	798	532
8	Hay-windrowed	478	287	191	Grapes_untrained	11271	6762	4,509	Self-blocking bricks	3682	2209	1473
9	Oats	20	12	8	Soil_vinyard_ develop	6203	3722	2481	Shadows	947	568	379
10	Soybeans-notill	972	583	389	Corn_senesced_green_weeds	3278	1967	1311				
11	Soybeans-mintill	2455	1473	982	Lettuce_romaine_4wk	1068	641	427				
12	Soybeans-clean	593	356	237	Lettuce_romaine_5wk	1927	1156	771				
13	Wheat	205	123	82	Lettuce_romaine_6wk	916	550	366				
14	Woods	1265	759	506	Lettuce_romaine_7wk	1070	642	428				
15	Buildings-grass-trees-drivers	386	231	155	Vinyard_untrained	7268	4361	2907				
16	Stone-steel-towers	93	56	37	Vinyard_vertical_trellis	1807	1084	723				

Total		10249	6150	4099		54129	32477	21652		42776	25666	17110

**Table 2:** Hyperparameter setting for the different tree-based ensemble classifiers.

Tree-based classifiers	Hyperparameter			
	Number of trees	Criterion for split quality	Maximum depth of tree	Maximum number of features for best split
DT	—	Entropy	50	20
ET	500	Entropy	50	20
RF	500	Gini	100	50
RoF	1000	Gini	100	50
RREF	1000	Gini	200	100

**Table 3:** Performance comparison between the oversampling techniques associated with different tree-based ensemble classifiers in terms of the percentages of precision, recall, *F* score, kappa, and OA and TE calculated in seconds for the IP dataset.

		Prec_score	Rec_score	*F*_score	Kappa_score	OA	TE (in secs)
DT	ROS	68.41 ± 1.8	67.13 ± 2.18	67.47 ± 2.61	44.62 ± 1.47	67.06 ± 1.74	3.516
	SMOTE	69.5 ± 2.49	68.77 ± 2.87	68.84 ± 2.95	45.92 ± 1.4	68.68 ± 1.93	4.629
	B-SMOTE	68.37 ± 1.6	67.51 ± 2.09	68.14 ± 2.19	45.89 ± 2.21	68.42 ± 1.83	6.495
	ADASYN	68.48 ± 2.65	67.74 ± 1.56	68.19 ± 2.35	45.41 ± 2.46	68.53 ± 1.38	7.895

ET	ROS	79.25 ± 2.12	80.61 ± 1.26	79.56 ± 1.39	60.43 ± 1.14	80.57 ± 1.24	6.439
	SMOTE	79.98 ± 2.68	81.25 ± 2.85	80.24 ± 2.13	62.3 ± 1.08	82.28 ± 1.76	7.867
	B-SMOTE	79.57 ± 1.58	81.16 ± 2.61	79.95 ± 3.02	60.76 ± 2.19	81.99 ± 1.25	9.137
	ADASYN	79.89 ± 2.28	80.93 ± 2.94	80.18 ± 1.44	62.21 ± 2.31	82.08 ± 1.09	9.248

RF	ROS	80.13 ± 1.12	81.15 ± 2.55	80.54 ± 1.28	71.8 ± 2.27	83.54 ± 1.89	6.256
	SMOTE	88.96 ± 1.12	81.72 ± 1.79	80.95 ± 1.15	72.98 ± 1.32	84.72 ± 1.12	8.282
	B-SMOTE	89.35 ± 1.76	80.91 ± 1.7	79.82 ± 2.51	61.86 ± 1.37	83.07 ± 1.2	10.056
	ADASYN	89.87 ± 1.63	81.33 ± 1.67	80.26 ± 2.34	72.05 ± 2.74	83.87 ± 1.78	10.01

RoF	ROS	84.32 ± 1.12	84.45 ± 1.11	84.5 ± 1.37	77.28 ± 1.87	85.15 ± 1.07	3274.945
	SMOTE	89.5 ± 2.07	89.53 ± 1.01	89.37 ± 1.06	81.34 ± 2.25	88.99 ± 1.85	3645.765
	B-SMOTE	88.16 ± 1.65	86.67 ± 2.73	7.39 ± 1.42	79.84 ± 2.4	87.65 ± 1.73	3958.983
	ADASYN	88.93 ± 2.61	88.56 ± 2.95	89.11 ± 2.47	80.37 ± 2.95	88.46 ± 1.68	3979.156

RREF	ROS	86.36 ± 1.31	87.92 ± 1.26	85.75 ± 2.57	79.84 ± 2.1	84.94 ± 1.09	43.875
	SMOTE	89.71 ± 2.46	90.15 ± 2.76	91.21 ± 1.1	83.48 ± 1.73	89.49 ± 1.32	56.953
	B-SMOTE	88.73 ± 2.92	88.94 ± 2.1	89.44 ± 2.94	79.8 ± 1.78	88.47 ± 1.94	55.326
	ADASYN	89.56 ± 2.2	89.85 ± 2.72	90.37 ± 1.02	80.36 ± 2.32	89.08 ± 1.53	61.578

**Table 4:** Performance comparison between the undersampling techniques associated with different tree-based ensemble classifiers in terms of the percentages of precision, recall, F score, kappa, and OA and TE calculated in seconds for the IP dataset.

		Prec_score	Rec_score	*F*_score	Kappa_score	OA	TE (in secs)
DT	RUS	53.16 ± 1.07	27.3 ± 1.35	26.27 ± 1.72	3.92 ± 1.68	27.35 ± 1.5	0.045
	Tomek	68.59 ± 1.09	68.44 ± 1.66	68.46 ± 1.69	44.14 ± 1.45	68.42 ± 1.58	2.93
	NCL	68.44 ± 1.35	74.74 ± 1.6	69.9 ± 1.46	44.68 ± 1.46	69.98 ± 1.04	2.079
	ENN	66.34 ± 1.3	67.51 ± 1.22	66.57 ± 1.57	43.46 ± 1.24	67.52 ± 2.21	1.16

ET	RUS	65.37 ± 1.66	38.29 ± 1.28	37.95 ± 1.09	38.95 ± 1.75	38.19 ± 2.83	0.216
	Tomek	78.32 ± 1.11	80.42 ± 1.15	78.89 ± 1.36	58.91 ± 1.53	79.95 ± 2.6	3.734
	NCL	75.17 ± 1.46	76.65 ± 1.39	75.28 ± 1.23	54.61 ± 1.95	76.6 ± 2.59	2.406
	ENN	72.3 ± 1.37	73.76 ± 1.29	72.24 ± 1.82	51.44 ± 1.41	73.75 ± 2.31	1.248

RF	RUS	64.84 ± 1.56	34.15 ± 1.27	32.23 ± 1.09	47.22 ± 1.76	34.06 ± 1.03	0.214
	Tomek	79.4 ± 1.73	80.69 ± 1.36	79.46 ± 1.15	61.25 ± 1.38	80.6 ± 1.58	4.297
	NCL	75.68 ± 1.04	77.1 ± 1.1	75.68 ± 1.43	56.29 ± 1	76.96 ± 2.72	3.557
	ENN	71.36 ± 1.37	72.84 ± 1.41	71.14 ± 1.63	51.55 ± 1.95	72.77 ± 2.54	0.515

RoF	RUS	67.87 ± 1.26	37.61 ± 1.13	38.62 ± 1.41	49.5 ± 1.35	40.84 ± 1.96	957.643
	Tomek	80.5 ± 1.34	82.14 ± 1.38	80.31 ± 1.49	76.35 ± 1.19	84.83 ± 2.08	1267.89
	NCL	74.33 ± 1.58	74.89 ± 1.15	74.29 ± 1.45	63.44 ± 1.4	79.25 ± 2.77	1043.895
	ENN	73.77 ± 1.31	72.61 ± 1.16	69.53 ± 1.1	59.83 ± 1.54	74.12 ± 1.68	996.054

RREF	RUS	69.11 ± 1.55	38.17 ± 1.46	39.21 ± 1.14	56.3 ± 1.04	44.37 ± 2.41	5.946
	Tomek	82.47 ± 1.24	82.61 ± 1.47	80.48 ± 1.37	78.58 ± 1.63	85.32 ± 2.76	9.279
	NCL	75.47 ± 1.23	75.97 ± 1.19	72.13 ± 1.66	62.1 ± 1.27	79.85 ± 1.15	6.789
	ENN	73.58 ± 1.62	71.51 ± 1.75	69.88 ± 1.28	60.62 ± 1	75.31 ± 2.09	6.241

**Table 5:** Performance comparison between the hybrid sampling techniques associated with different tree-based ensemble classifiers in terms of the percentages of precision, recall, F score, kappa, and OA and TE calculated in seconds for the IP dataset.

		Prec_score	Rec_score	*F*_score	Kappa_score	OA	TE (in secs)
DT	SMOTETomek	68.39 ± 1.77	68.46 ± 1.13	68.37 ± 1.46	45.21 ± 1.92	68.49 ± 2.38	16.956
	SMOTEENN	66.16 ± 1.35	67.14 ± 1.48	66.31 ± 1.72	46.78 ± 1.5	67.134 ± 2.18	16.357

ET	SMOTETomek	78.56 ± 1.2	80.15 ± 1.57	79.09 ± 1.51	59.69 ± 1.76	80.09 ± 1.34	15.672
	SMOTEENN	72.55 ± 1.04	74.2 ± 1.39	72.44 ± 1.84	52.31 ± 1.48	73.96 ± 2.31	15.118

RF	SMOTETomek	79.12 ± 1.26	80.55 ± 1.71	79.46 ± 1.32	61.37 ± 1.17	80.54 ± 2.19	18.171
	SMOTEENN	72.13 ± 1.82	73.36 ± 1.72	71.64 ± 1.99	52.67 ± 1.55	73.317 ± 2.33	16.613

RoF	SMOTETomek	82.37 ± 1.12	84.23 ± 1.66	81.56 ± 1.12	64.74 ± 1.17	82.74 ± 2.16	4195.326
	SMOTEENN	74.66 ± 1.58	75.82 ± 1.75	73.73 ± 1.78	54.85 ± 1.43	76.19 ± 1.73	3628.153

RREF	SMOTETomek	81.71 ± 1.02	81.13 ± 1.59	79.93 ± 1.44	62.83 ± 1.6	82.32 ± 1.36	89.452
	SMOTEENN	74.12 ± 1.29	74.29 ± 1.85	73.16 ± 1.67	53.19 ± 1.56	75.43 ± 1.01	85.31

**Table 6:** Performance comparison between the oversampling techniques associated with different tree-based ensemble classifiers in terms of the percentages of precision, recall, F score, kappa, and OA and TE calculated in seconds for the SV dataset.

		Prec_score	Rec_score	*F*_score	Kappa_score	OA	TE (in secs)
DT	ROS	88.51 ± 2.86	89.54 ± 2.75	89.59 ± 2.46	42.17 ± 2.57	88.78 ± 1.74	27.487
	SMOTE	89.48 ± 1.38	89.49 ± 1.87	90.91 ± 2.56	46.63 ± 2.17	89.36 ± 1.19	32.258
	B-SMOTE	89.47 ± 2.45	89.39 ± 1.95	89.47 ± 2.75	46.22 ± 2.11	89.05 ± 1.35	37.898
	ADASYN	89.64 ± 2.43	89.62 ± 1.84	89.67 ± 1.3	48.3 ± 2.06	89.59 ± 1.63	39.618

ET	ROS	93.36 ± 2.71	93.33 ± 2.31	93.32 ± 2.22	60.78 ± 2.02	92.05 ± 1.84	51.385
	SMOTE	93.35 ± 2.82	93.29 ± 2.2	93.24 ± 2.1	62.69 ± 2.72	93.11 ± 1.22	52.384
	B-SMOTE	93.36 ± 1.41	93.32 ± 1.86	93.34 ± 2.45	61.88 ± 1.57	92.67 ± 1.47	64.989
	ADASYN	93.46 ± 2.86	93.35 ± 2.13	93.48 ± 2.86	62.92 ± 2.5	93.3 ± 1.13	65.096

RF	ROS	93.31 ± 2.29	93.26 ± 2.9	93.29 ± 1.11	59.76 ± 2.78	92.26 ± 1.23	67.545
	SMOTE	93.36 ± 1.15	93.32 ± 1.47	93.26 ± 2.09	61.84 ± 1.86	93.27 ± 1.7	68.773
	B-SMOTE	93.11 ± 2.21	93.6 ± 2.02	93.23 ± 1.96	60.44 ± 2.31	93.04 ± 1.41	68.56
	ADASYN	93.48 ± 1.37	93.32 ± 1.58	93.31 ± 2.99	62.88 ± 2.05	94.64 ± 1.36	68.796

RoF	ROS	94.47 ± 1.04	94.55 ± 1.78	94.52 ± 1.83	71.79 ± 2.77	94.85 ± 1.23	4644.89
	SMOTE	95.43 ± 1.72	95.56 ± 2.75	95.27 ± 1.35	72.36 ± 1.68	95.48 ± 1.27	4889.134
	B-SMOTE	95.48 ± 2.59	95.59 ± 2.46	95.25 ± 1.31	72.51 ± 1.51	95.18 ± 1.36	4755.675
	ADASYN	95.6 ± 1.8	95.87 ± 1.57	95.75 ± 2.84	72.68 ± 2.89	95.7 ± 1.86	5023.45

RREF	ROS	94.23 ± 2.27	94.07 ± 2.63	94.59 ± 2.94	81.86 ± 1.41	93.27 ± 1.42	75.543
	SMOTE	94.25 ± 1.16	93.97 ± 2.59	94.3 ± 2.78	81.12 ± 1.4	95.57 ± 1.18	87.642
	B-SMOTE	94.47 ± 2.18	92.45 ± 2.32	94.21 ± 1.79	80.12 ± 2.07	94.25 ± 1.13	89.654
	ADASYN	95.43 ± 1.45	94.83 ± 1.85	94.82 ± 2.7	82.94 ± 1.02	95.84 ± 1.33	95.437

**Table 7:** Performance comparison between the undersampling techniques associated with different tree-based ensemble classifiers in terms of the percentages of precision, recall, F score, kappa, and OA and TE calculated in seconds for the SV dataset.

		Prec_score	Rec_score	*F*_score	Kappa_score	OA	TE (in secs)
DT	RUS	82.74 ± 2.98	76.44 ± 2.5	77.23 ± 1.49	39.94 ± 2.09	76.4 ± 1.47	2.067
	Tomek	89.81 ± 2.48	89.42 ± 2.91	89.32 ± 2.68	46.75 ± 1.65	79.83 ± 1.06	3.705
	NCL	89.86 ± 1.99	89.89 ± 2.05	89.87 ± 2.77	46.17 ± 1.64	79.97 ± 1.19	2.13
	ENN	89.3 ± 2.75	88.94 ± 2.78	88.98 ± 2.48	45.7 ± 2.47	78.93 ± 1.15	3.545

ET	RUS	86.91 ± 2.42	82.9 ± 1.15	83.45 ± 2.19	58.68 ± 2.11	82.95 ± 2.69	3.256
	Tomek	93 ± 2.55	93.9 ± 2.56	93.6 ± 1.02	60.42 ± 2.16	93.22 ± 1.24	19.895
	NCL	91.51 ± 2.4	91.45 ± 1.62	91.35 ± 2.77	58.67 ± 1.93	91.36 ± 1.72	18.444
	ENN	90.67 ± 1.24	90.46 ± 1.99	90.45 ± 1.53	57.58 ± 1.8	90.44 ± 1.32	18.404

RF	RUS	87.12 ± 1.14	83.35 ± 2.74	83.8 ± 1.31	69.4 ± 2.46	83.35 ± 1.15	3.244
	Tomek	93.12 ± 2.37	93.18 ± 2.65	93.35 ± 1.47	70.31 ± 1.95	93.56 ± 1.25	29.694
	NCL	91.68 ± 2.89	91.55 ± 1.54	91.55 ± 1.77	68.73 ± 2.06	91.49 ± 2.12	28.271
	ENN	90.57 ± 1.6	90.43 ± 2.9	90.48 ± 2.4	67.49 ± 1.06	90.41 ± 1.73	27.399

RoF	RUS	88.77 ± 1.94	83.54 ± 2.24	84.1 ± 2.73	80.1 ± 1.53	85.32 ± 1.81	103.763
	Tomek	93.44 ± 2.83	94.8 ± 1.99	94.92 ± 1.65	81.45 ± 1.58	94.41 ± 1.5	3219.078
	NCL	92.26 ± 2.34	93.23 ± 2.57	93.37 ± 2.42	80.27 ± 2.38	93.68 ± 2.84	2964.741
	ENN	90.29 ± 2.73	92.32 ± 2.82	91.7 ± 2.49	79.17 ± 2.59	93.54 ± 2.98	2895.973

RREF	RUS	88.65 ± 1.03	81.8 ± 2.19	79.79 ± 1.84	79.62 ± 2.09	84.93 ± 2	19.439
	Tomek	93.88 ± 2.39	94.48 ± 2.57	94.94 ± 1.32	81.59 ± 2.63	94.44 ± 2.31	25.452
	NCL	91.82 ± 1.58	92.26 ± 2.15	91.32 ± 1.41	78.8 ± 2.21	93.7 ± 2.6	21.214
	ENN	89.88 ± 1.98	90.68 ± 2.52	91.94 ± 2.65	79.11 ± 1.46	93.18 ± 2.41	20.84

**Table 8:** Performance comparison between the hybrid sampling techniques associated with different tree-based ensemble classifiers in terms of the percentages of precision, recall, F score, kappa, and OA and TE calculated in seconds for the SV dataset.

		Prec_score	Rec_score	*F*_score	Kappa_score	OA	TE (in secs)
DT	SMOTETomek	89.55 ± 2.04	89.54 ± 1.64	89.58 ± 1.16	46.28 ± 1.87	89.46 ± 2.11	91.796
	SMOTEENN	88.72 ± 2.24	88.62 ± 1.54	88.6 ± 2.53	45.18 ± 2.76	88.59 ± 1.95	83.808

ET	SMOTETomek	93.12 ± 2.67	93.19 ± 1.61	93.12 ± 1.21	60.34 ± 2.82	93.01 ± 2.35	105.412
	SMOTEENN	90.64 ± 2.36	90.41 ± 1.14	90.49 ± 1.71	57.31 ± 1.96	90.38 ± 1.87	99.811

RF	SMOTETomek	93.01 ± 2.42	93.28 ± 2.9	93.29 ± 1.32	68.35 ± 1.15	92.97 ± 2.14	59.343
	SMOTEENN	90.59 ± 1.96	90.31 ± 1.25	90.21 ± 2.06	67.27 ± 2.51	90.26 ± 1.84	52.498

RoF	SMOTETomek	94.89 ± 1.39	94.77 ± 1.74	94.81 ± 1.54	79.28 ± 1.03	93.89 ± 2.24	6297.542
	SMOTEENN	91.72 ± 1	92.12 ± 1.26	91.94 ± 1.18	69.11 ± 1.81	91.77 ± 1.68	6031.47

RREF	SMOTETomek	93.29 ± 2.83	93.14 ± 2.09	93.57 ± 1.26	78.49 ± 2.07	93.17 ± 2.12	186.743
	SMOTEENN	91.09 ± 1.12	91.17 ± 1.54	91.79 ± 1.46	88.2 ± 1.52	91.41 ± 1.48	163.265

**Table 9:** Performance comparison between the oversampling techniques associated with different tree-based ensemble classifiers in terms of the percentages of precision, recall, F score, kappa, and OA and TE calculated in seconds for the PU dataset.

		Prec_score	Rec_score	*F*_score	Kappa_score	OA	TE (in secs)
DT	ROS	78.96 ± 1.91	79.28 ± 2.13	79.1 ± 3.36	37.12 ± 2.93	77.17 ± 1.86	28.216
	SMOTE	79.45 ± 2.14	79.96 ± 2.61	79.89 ± 1.47	37.92 ± 1.22	79.94 ± 1.17	29.025
	B-SMOTE	78.73 ± 3.17	78.43 ± 3.04	78.62 ± 3.33	35.15 ± 3.54	78.37 ± 1.06	28.815
	ADASYN	78.63 ± 2.27	78.12 ± 3.04	78.41 ± 2.54	35.45 ± 1.9	78.57 ± 1.7	30.451

ET	ROS	84.79 ± 2.64	85.77 ± 1.35	83.58 ± 3.44	43.85 ± 2.98	84.67 ± 1.95	56.781
	SMOTE	84.93 ± 2.65	85.86 ± 3.15	83.69 ± 2.74	45.9 ± 1.26	85.71 ± 1.59	57.864
	B-SMOTE	84.74 ± 3.2	85.72 ± 1.33	83.55 ± 3.84	44.82 ± 2.91	85.17 ± 1.89	57.336
	ADASYN	84.61 ± 1.1	85.69 ± 2.51	83.43 ± 1.87	44.27 ± 2.12	85.51 ± 1.8	59.337

RF	ROS	84.28 ± 2.66	85.26 ± 3.95	83.15 ± 2.39	45.24 ± 3.28	84.25 ± 1.65	62.336
	SMOTE	84.56 ± 1.41	85.96 ± 2.73	83.8 ± 3.6	48.04 ± 1.31	86.89 ± 1.97	65.369
	B-SMOTE	83.17 ± 3.28	85.9 ± 1.48	81.9 ± 1.82	37.28 ± 2.41	84.01 ± 1.43	65.57
	ADASYN	83.92 ± 2.99	84.82 ± 2.87	81.94 ± 1.13	38.87 ± 3.27	86.04 ± 1.88	66.634

RoF	ROS	86.57 ± 1.34	86.85 ± 3.49	84.96 ± 2.48	50.25 ± 3.32	87.24 ± 1.25	3982.435
	SMOTE	87.93 ± 3.64	88.26 ± 3.12	88.17 ± 2.29	52.93 ± 1.2	91.89 ± 1.45	3543.257
	B-SMOTE	87.52 ± 3.56	88.22 ± 1.28	88.03 ± 2.55	43.48 ± 1.47	88.55 ± 1.56	3421.805
	ADASYN	87.49 ± 1.93	88.1 ± 3.46	88.1 ± 3.64	40.16 ± 2.83	90.38 ± 1.1	4721.63

RREF	ROS	86.59 ± 3.82	86.83 ± 3.38	84.99 ± 3.65	50.28 ± 3.83	87.24 ± 1.4	85.87
	SMOTE	88.96 ± 3.32	88.79 ± 1.83	88.95 ± 1.46	52.32 ± 1.5	91.98 ± 1.16	87.654
	B-SMOTE	87.54 ± 3.74	88.2 ± 3.61	88.09 ± 2.17	43.48 ± 2.71	88.53 ± 1.83	84.764
	ADASYN	87.45 ± 3.83	88.19 ± 1.18	88.17 ± 2.92	51.24 ± 3.07	90.58 ± 1.74	88.453

**Table 10:** Performance comparison between the undersampling techniques associated with different tree-based ensemble classifiers in terms of the percentages of precision, recall, F score, kappa, and OA and TE calculated in seconds for the PU dataset.

		Prec_score	Rec_score	F_score	Kappa_score	OA	TE (in secs)
DT	RUS	78.92 ± 1.16	43.2 ± 1.55	48.35 ± 2.9	15.29 ± 1.57	42.96 ± 1.85	0.818
	Tomek	78.76 ± 2.81	79.17 ± 1.14	78.99 ± 2.88	36.7 ± 1.26	79.13 ± 1.79	3.185
	NCL	79.82 ± 1.46	81.79 ± 2.49	80.36 ± 1.77	39.89 ± 1.86	82.51 ± 2.35	2.588
	ENN	79.38 ± 2.85	81.53 ± 1.71	79.78 ± 2.13	39.82 ± 2.86	81.69 ± 1.91	2.566

ET	RUS	83.24 ± 2.33	44.17 ± 2.06	48.14 ± 2.15	17.74 ± 1.15	44.06 ± 2.5	11.806
	Tomek	84.4 ± 2.39	85.53 ± 2.64	83.22 ± 2.91	42.91 ± 2.15	85.49 ± 1.84	14.223
	NCL	83.87 ± 1.59	84.96 ± 1.93	82.57 ± 1.28	42.25 ± 2.88	84.92 ± 2.2	13.34
	ENN	82.83 ± 1.8	84.11 ± 1.91	81.36 ± 2.98	37.48 ± 1.49	84.13 ± 1.81	13.806

RF	RUS	83.36 ± 2.52	43.2 ± 1.51	47.17 ± 1.63	17.16 ± 1.79	43.16 ± 2.71	23.161
	Tomek	84.19 ± 1.31	85.33 ± 1.05	83.12 ± 1.17	43.17 ± 1.82	85.29 ± 2.17	28.126
	NCL	83.75 ± 1.55	84.91 ± 2.43	82.7 ± 2.23	42.84 ± 2.25	84.97 ± 2.67	27.781
	ENN	82.81 ± 2.1	84.12 ± 1.68	81.43 ± 1.75	38.27 ± 1.56	84.1 ± 2.17	27.765

RoF	RUS	85.32 ± 1.64	48.46 ± 1.66	49.77 ± 2.67	19.87 ± 2.67	47.29 ± 1.11	1769.731
	Tomek	85.97 ± 2.34	86.34 ± 1.44	85.34 ± 2.43	47.52 ± 1.35	88.71 ± 1.34	2135.432
	NCL	84.31 ± 2.23	85.81 ± 2.26	83.2 ± 1.91	47.63 ± 2.58	87.43 ± 2.58	1960.372
	ENN	83.11 ± 1.02	84.59 ± 2.82	81.8 ± 2.73	41.22 ± 3	87.11 ± 1.75	1904.97

RREF	RUS	85.13 ± 1.38	47.71 ± 2.68	49.28 ± 2.9	18.85 ± 1.96	46.85 ± 1.16	17.298
	Tomek	85.33 ± 1.22	85.95 ± 1.64	85.81 ± 2.47	46.24 ± 1.09	89.92 ± 2.11	23.064
	NCL	84.02 ± 1.8	85.49 ± 1.29	83.69 ± 2.27	45.87 ± 2.26	87.81 ± 2.32	21.873
	ENN	82.91 ± 1.57	84.8 ± 2.38	82.24 ± 1.6	39.97 ± 2.78	87.03 ± 1.84	20.09

**Table 11:** Performance comparison between the hybrid sampling techniques associated with different tree-based ensemble classifiers in terms of the percentages of precision, recall, F score, kappa, and OA and TE calculated in seconds for the PU dataset.

		Prec_score	Rec_score	F_score	Kappa_score	OA	TE (in secs)
DT	SMOTETomek	78.77 ± 1.32	78.93 ± 2.3	78.82 ± 2.93	35.37 ± 1.11	78.89 ± 2.51	89.671
	SMOTEENN	79.45 ± 2.63	81.53 ± 2.85	79.88 ± 2.93	37.62 ± 2.05	81.47 ± 2.18	80.852

ET	SMOTETomek	84.46 ± 2.78	85.47 ± 1.67	83.18 ± 1.18	42.71 ± 1.04	85.36 ± 1.34	97.329
	SMOTEENN	82.91 ± 1.23	84.17 ± 2.25	81.42 ± 1.85	39.77 ± 1.32	84.12 ± 2.45	94.81

RF	SMOTETomek	82.95 ± 2.23	83.85 ± 1.69	80.74 ± 1.25	48.99 ± 1.6	83.84 ± 2.67	101.411
	SMOTEENN	82.61 ± 1.08	83.73 ± 2.88	80.72 ± 1.18	48.68 ± 2.66	83.73 ± 2.71	96.546

RoF	SMOTETomek	85.28 ± 2.34	86.95 ± 2.75	87.37 ± 1.95	66.42 ± 2.64	86.12 ± 2.14	5231.754
	SMOTEENN	85.18 ± 1.69	86.71 ± 2.51	87.48 ± 1.51	49.88 ± 2.68	85.54 ± 1.07	4987.34

RREF	SMOTETomek	84.79 ± 1.8	85.7 ± 2.7	86.52 ± 1.75	78.13 ± 1.14	85.83 ± 2.32	162.864
	SMOTEENN	84.44 ± 2.29	85.27 ± 2.97	85.74 ± 2.08	50.49 ± 2.93	85.17 ± 1.33	149.063

## Data Availability

Publicly available data are used in this study.

## Abbreviations

HS: Hyperspectral
HSI: Hyperspectral image
GIS: Geographic information system
PCA: Principal component analysis
ML: Machine learning
DL: Deep learning
IP: Indian Pines
SV: Salinas Valley
UP: University of Pavia
LR: Logistic regression
NB: Naive Bayes
*k*-NN: *K*-nearest neighbor
SVM: Support vector machine
DT: Decision tree
ET: Extra trees or extremely randomized trees
RF: Random forest
RoF: Rotation forest
RREF: Random rotation ensemble forest
ROS: Random oversampling
SMOTE: Synthetic minority oversampling technique
B-SMOTE: Borderline synthetic minority oversampling technique
ADASYN: Adaptive synthetic minority oversampling technique
RUS: Random undersampling
TLs: Tomek Links
NCL: Neighborhood cleaning rule
ENN: Edited nearest neighbor
CART: Classification and regression tree.
